# Plasma Pharmacokinetic Determination of Canagliflozin and Its Metabolites in a Type 2 Diabetic Rat Model by UPLC-MS/MS

**DOI:** 10.3390/molecules23051229

**Published:** 2018-05-20

**Authors:** Song-tao Dong, Hui-min Niu, Yin Wu, Jia-lei Jiang, Ying Li, Kun-yu Jiang, Xin Wang, Mao-fan Zhang, Ming-feng Han, Sheng-nan Meng

**Affiliations:** 1 Department of Pharmaceutics, School of Pharmacy, China Medical University, Shenyang 110001, China; dongsongtao8886@163.com (S.-t.D.); 15804066249@163.com (H.-m.N.); 15804002893@163.com (J.-l.J.); jiangkunyu87@163.com (K.-y.J.); WangXinCMU@126.com (X.W.); mfzhang@cmu.edu.cn (M.-f.Z.); 18698857919@163.com (M.-f.H.); 2 Department of Pharmaceutics, School of Pharmacy, Hebei Medical University, Shijiazhuang 050017, China; wuyin82@126.com (Y.W.); lyyaoda@126.com (Y.L.)

**Keywords:** canagliflozin, metabolites, diabetes, UPLC-MS/MS

## Abstract

Canagliflozin is a novel, orally selective inhibitor of sodium-dependent glucose co-transporter-2 (SGLT2) for the treatment of patients with type 2 diabetes mellitus. In this study, a sensitive and efficient UPLC-MS/MS method for the quantification of canagliflozin and its metabolites in rat plasma was established and applied to pharmacokinetics in a type 2 diabetic rat model. We firstly investigated the pharmacokinetic changes of canagliflozin and its metabolites in type 2 diabetic rats in order to use canagliflozin more safely, reasonably and effectively. We identified three types of O-glucuronide metabolites (M5, M7 and M17), two kinds of oxidation metabolites (M8 and M9) and one oxidation and glucuronide metabolite (M16) using API 5600 triple-TOF-MS/MS. Following liquid–liquid extraction by tert-butyl methyl ether, chromatographic separation of canagliflozin and its metabolites were performed on a Waters XBridge BEH C18 column (100 × 2.1 mm, 2.5 μm) using 0.1% acetonitrile–formic acid (75:15, *v*/*v*) as the mobile phase at a flow rate of 0.7 mL/min. Selected ion monitoring transitions of *m*/*z* 462.00→191.10, 451.20→153.10, 638.10→191.10 and 478.00→267.00 were chosen to quantify canagliflozin, empagliflozin (IS), O-glucuronide metabolites (M5, M7 and M17), and oxidation metabolites (M9) using an API 5500-triple-MS/MS in the positive electrospray ionization mode. The validation of the method was found to be of sufficient specificity, accuracy and precision. The pathological condition of diabetes could result in altered pharmacokinetic behaviors of canagliflozin and its metabolites. The pharmacokinetic parameters (AUC_0–t_, AUC_0–∞_, CL_z_/F, and V_z_/F) of canagliflozin were significantly different between the CTRL and DM group rats (*p* < 0.05 or *p* < 0.01), which may subsequently cause different therapeutic effects.

## 1. Introduction

Type 2 diabetes mellitus (T2DM), a worldwide chronic disease accounting for more than 90% of all diabetic patients, is characterized by hyperglycemia due to a deficiency in insulin secretion, excessive hepatic glucose production and insulin resistance [1,2]. According to statistics, the global prevalence of T2DM is projected to increase to 522 million by 2030 [3]. Sodium glucose cotransporter 2 (SGLT2), which is primarily located in the early proximal renal tubule, is responsible for approximately 90% of the glucose reabsorption in the kidney [4]. Therefore, inhibition of SGLT2 can increase glucose excretion and decrease glucose reabsorption in renal tubules, thereby reducing the blood glucose level and improving insulin resistance in T2DM patients [5].

As an active inhibitor of SGLT2, canagliflozin, a novel oral hypoglycemic agent, has been approved as an adjunct to diet and exercise to improve glycemic control in T2DM patients in several countries [6,7,8]. By inhibiting the expression of SGLT2 in the proximal renal tubules, canagliflozin can reduce the reabsorption of filtered glucose, thereby increasing urinary glucose excretion (UGE). Results of preclinical studies and randomized clinical trials (RCTs) have shown that canagliflozin can reduce renal glucose reabsorption, increase urinary glucose excretion, reduce plasma glucose, and promote weight loss [9]. Meanwhile, canagliflozin can also be combined with current hypoglycemic drugs, including metformin [10], pioglitazone [11], insulin [12] and so on. In addition, placebo-controlled cardiovascular outcome trail of canagliflozin (CANVAS-REG OUTCOME) results have translated into the recognition of canagliflozin as a method of cardiovascular protective therapy for patients with diabetes by the US Food and Drug Administration, the American College of Cardiology/American Heart Association, Diabetes Canada and the European Society of Cardiology. It has been shown that canagliflozin has multiple therapeutic effects in clinical therapy.

**Figure 1:**
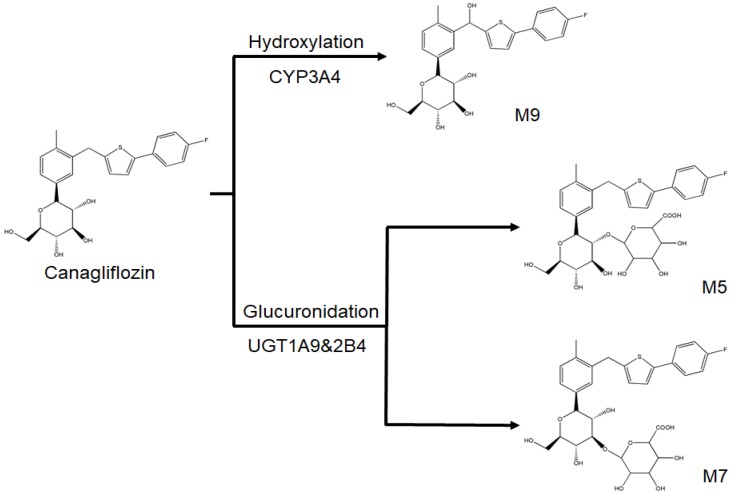
Metabolism pathway for canagliflozin.

Canagliflozin was extensively metabolized via O-glucuronidation by the enzymes uridine diphosphate-glucuronosyltransferase 1A9 (UGT1A9) and UGT2B4 to O-glucuronide metabolites, M5 and M7, and to a smaller extent by cytochrome P450 (CYP3A4) (M9) (Figure 1) [13]. Inducers of CYP and UGT enzymes (e.g., rifampicin, phenytoin, barbiturates, etc) can decrease the plasma concentration of canagliflozin in patients and healthy participants, which may decrease the efficacy of canagliflozin [14]. In addition, the pharmacokinetics of major metabolites can clearly reflect the metabolism procession of canagliflozin in vivo [15,16]. Therefore, it is very important for us to simultaneously explore the metabolism pathways of canagliflozin and its metabolites in vivo in order to use the drug more safely, reasonably, and effectively. In addition, a sensitive and effective quantification analysis method for canagliflozin and its metabolites is the basis of drug metabolism pathway research in vivo. However, there are many analysis methods for canagliflozin [15,17,18] and few studies have focused on the drug and its metabolites simultaneously.

In the present study, we developed a sensitive and effective UPLC-MS/MS quantification and semi-quantification method to simultaneously detect canagliflozin and its metabolites in rat plasma, respectively. Meanwhile, we reported the pharmacokinetics of canagliflozin and transformations of its four main metabolites in type 2 diabetic model rats, and these alternations may have an effect on the application of canagliflozin in clinical therapy.

## 2. Results and Discussion

### 2.1. Identification of Canagliflozin and Its Metabolites by UPLC-TOF-MS/MS

**Figure 2:**
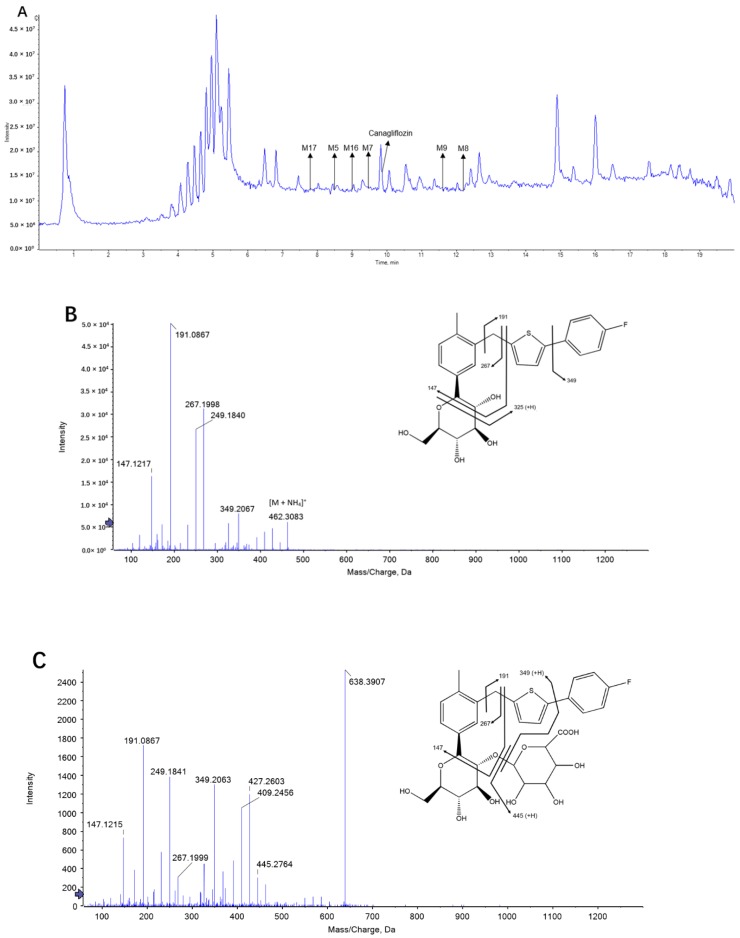

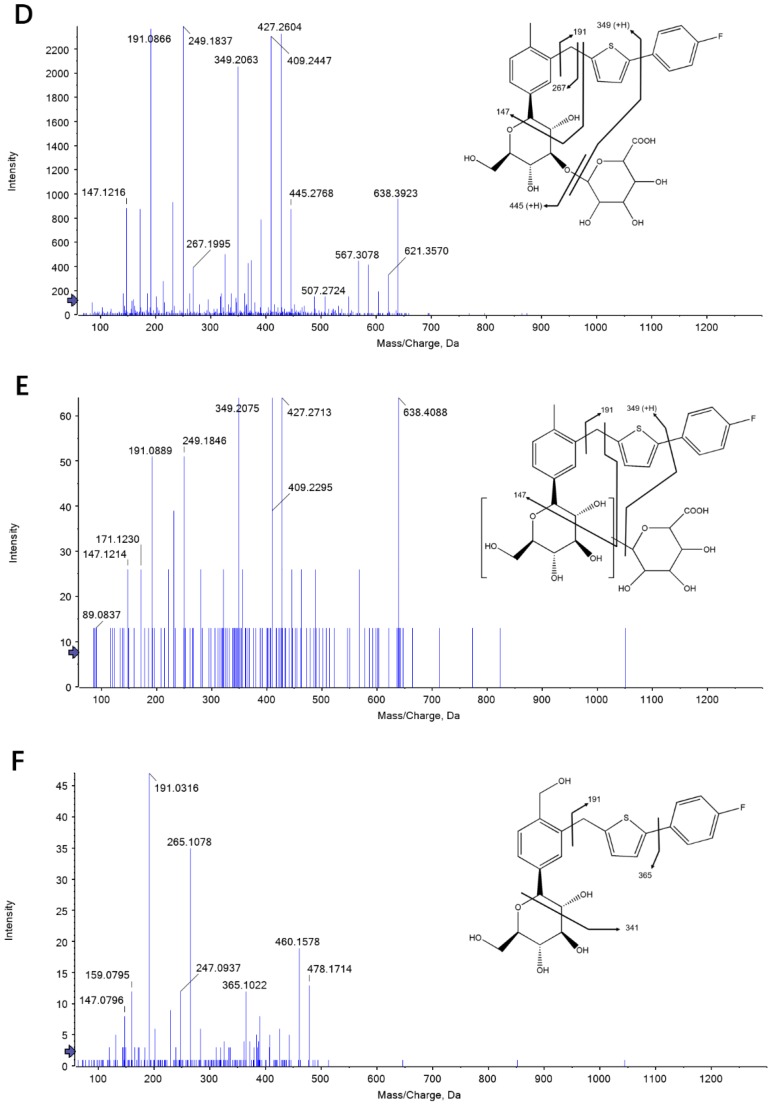

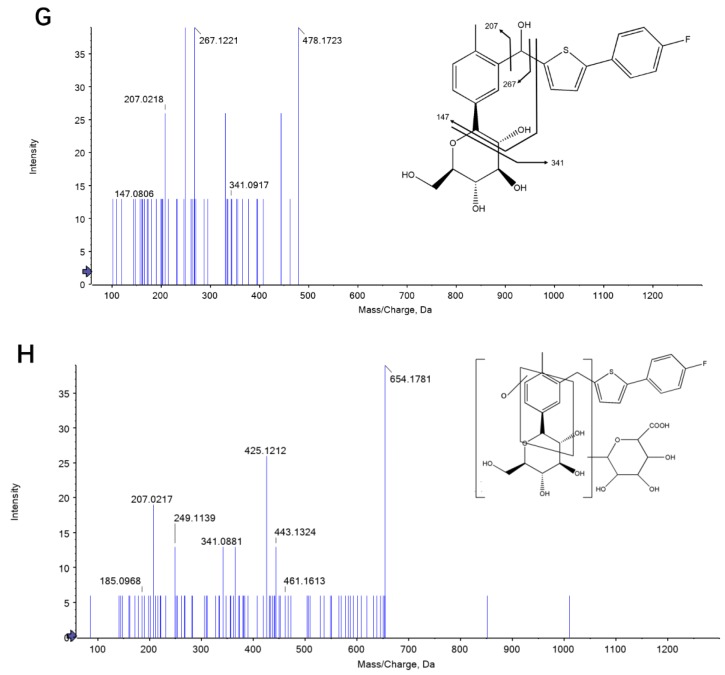
The base peak chromatogram and product ion mass spectra of canagliflozin (**B**), M5 (**C**), M7 (**D**), M17 (**E**), M8 (**F**), M9 (**G**) and M16 (**H**).

**Figure 3:**
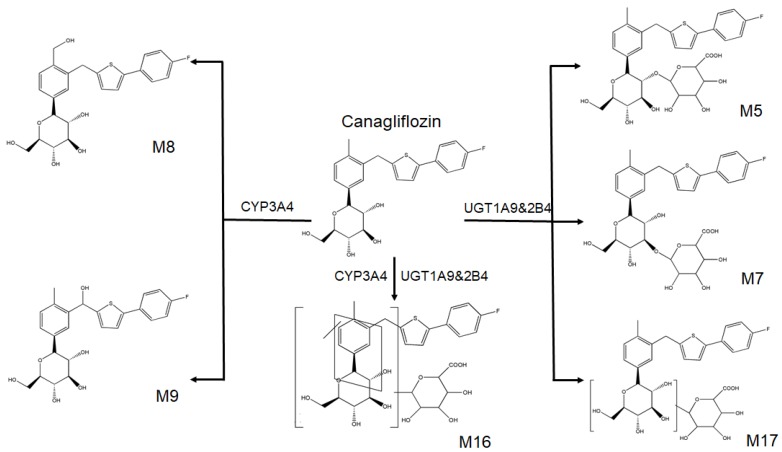
Proposed metabolic pathways for canagliflozin in rats.

Canagliflozin and its metabolites (M5, M7, M17, M8, M9, M16) were initially identified by UPLC-triple-TOF-MS/MS in rat plasma (Figure 2). In addition, we detected three O-glucuronide metabolites (M5, M7 and M17), two kinds of oxidation metabolites (M8 and M9) and one oxidation and glucuronide metabolite (M16). The proposed metabolic pathways for canagliflozin in rat plasma are shown in Figure 3.

The development of analytical techniques, especially UPLC-MS/MS, has allowed intensive study about metabolites of drugs in vivo [19,20,21]. The main advantage of UPLC-TOF-MS/MS is the ability to analysis metabolites systematically and comprehensively. The analysis parameters and product ion mass spectra of canagliflozin and its metabolites could both help us to explore the UPLC-MS/MS quantification analysis method. We found that the concentrations of M5, M7, M9 and M17 are high in rat plasma and that M5, M7 and M9 are the main metabolites of canagliflozin. However, the concentrations of M8 and M16 were almost too low to quantify in rat plasma. Therefore, we established a UPLC-MS/MS analysis method for canagliflozin, M5, M7, M9 and M17 to explore the metabolic procession of canagliflozin in type 2 diabetic rats.

### 2.2. UPLC-MS/MS Quantification Method

#### 2.2.1. Optimization of UPLC-MS/MS Parameters

**Figure 4:**
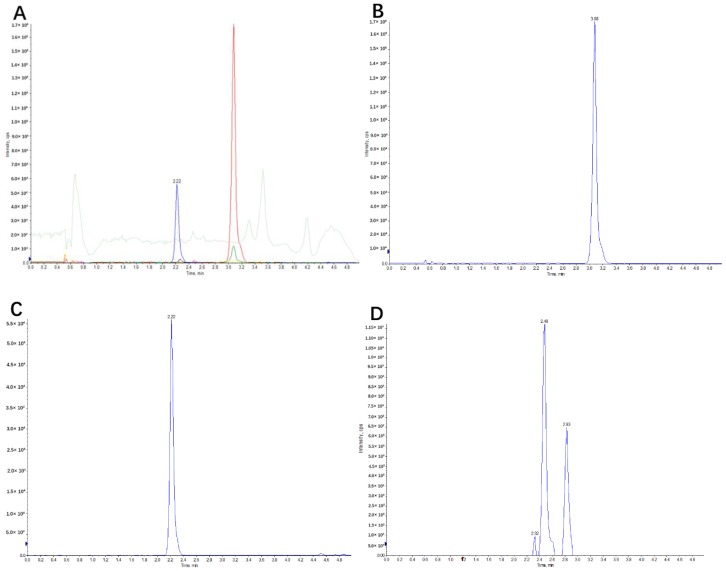

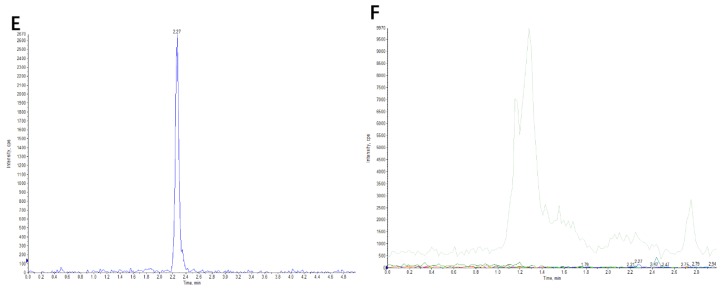
Typical multiple reaction monitoring (MRM) chromatograms of (**A**) the base peak chromatogram; (**B**) canagliflozin; (**C**) the internal standard (empagliflozin); (**D**) the O-glucuronide metabolites, M5, M7 and M17; (**E**) the oxidation metabolite, M9; and (**F**) blank plasma.

Based on the results of UPLC-TOF-MS/MS, we investigated a reliable and selective quantification and semi-quantification analysis method for canagliflozin and its metabolites, respectively. In the initial step of method development, both positive and negative ESI (Electrospray ionization) modes were used to detect the MS spectra of canagliflozin. The positive ion [M + NH_4_]^+^ resulted in a stronger signal intensity, and then the ion source was run in positive mode. The multiple reaction monitoring (MRM) transitions, *m*/*z* 462.00→191.10, 451.20→153.10, 638.10→191.10 and 478.00→267.00, were chosen to quantify canagliflozin, empagliflozin (IS), O-glucuronide metabolites (M5, M7 and M17), and an oxidation metabolite (M9), respectively. The MS/MS spectra of canagliflozin, IS and its metabolites are shown in Figure 4. (Supplementary material Figure S1 ).

The total assay time was kept within 5.1 min and the gradient schedules were sharp and symmetrical under the chromatographic conditions. Under the established elution conditions, canagliflozin, empagliflozin, O-glucuronide metabolites (M5, M7, M17) and the oxidation metabolite (M9) eluted at 3.08 min, 2.2 min, (2.32 min, 2.48 min and 2.83 min) and 2.27 min, respectively. No significant interference was observed.

#### 2.2.2. Linearity, Sensitivity and Specificity

Calibration standards were prepared and analyzed at eight different concentrations, from 10 to 1000 ng/mL. The calibration curve for canagliflozin displayed a good linear relationship in the concentration range *Y* = 0.00575*X* + 0.0214. The LLOQ (Lower limit of quantitation) of the developed assay for canagliflozin was 10 ng/mL. Selected reaction monitoring chromatograms of blank rat plasma, and the quality control samples of canagliflozin at concentrations of 800 ng/mL (high), 500 ng/mL (medium), 25 ng/mL (low) and 10 ng/mL (LLOQ) were performed. No interference at the retention times of canagliflozin (~3.08 min) or IS (~2.22 min) in any MRM chromatograms of blank plasma samples was observed. Canagliflozin at high, medium, low and LLOQ concentrations was clearly detected. No interference at the retention time of canagliflozin or IS were observed for the plasma samples obtained from rat plasma after oral administration of canagliflozin, indicating good selectivity and specificity.

#### 2.2.3. Accuracy, Precision and Matrix Effect

The intra-day and inter-day accuracy and precision values are shown in Table 1. A high concentration (800 ng/mL), a medium concentration (500 ng/mL), a low concentration (25 ng/mL) and LLOQ (10 ng/mL) QC (Quality control) samples were used for the analysis. The intra-day and inter-day precision values were detected with RSD (Relative standard deviation), ranging from 1.95 to 9.64% (intra-day) and 1.65–10.68% (inter-day) and ranged within the acceptance limit of 15%. These results demonstrate that this assay possesses good accuracy and precision for detecting canagliflozin in rat plasma. The matrix effects for canagliflozin and IS were in the range of 85% to 115%, which indicated that the matrix effects did not significantly interfere with canagliflozin determination in rat blood.

#### 2.2.4. Stability

High, medium and low levels of QC concentrations were used to evaluate the stability of canagliflozin in plasma under the experimental conditions. As shown in Table 2, canagliflozin was found to be stable in rat plasma at room temperature (25 °C) for 24 h. Canagliflozin also shown good stability after 24 h in the auto-sampler temperature (4 °C) and at −80 °C followed by exposure to three freeze and thaw cycles. In addition, the canagliflozin was found to be stable under the storage conditions (−80 °C) for at least 30 days.

### 2.3. Pharmacokinetics in Type 2Diabetic Rats

Parameters such as triglyceride, total cholesterol, blood glucose, homeostasis model assessment of insulin resistance (HOMA-IR) and body weight were measured in the CTRL rats and DM rats (Table 3). The blood levels of glucose, triglycerides, and total cholesterol in the DM rats were markedly higher than those in the CTRL rats. DM rats also had significantly increased HOMA-IR values, accompanied by reduced body weight (Table 3) and the development of diabetic symptoms, such as polyuria, polyphagia and polydipsia. These indexes were similar to those of type 2 diabetic patients, indicating that the DM rats may be considered to be type 2 diabetic rats [22].

In our study, we oral administered 20 mg/kg of canagliflozin to rats by gavage. The dose was based on the clinical daily dose of canagliflozin as well as published reports about the biological characteristics of canagliflozin [23,24,25]. In addition, we referred to published articles about the pharmacokinetics of canagliflozin in rats [26] and previous results indicated that the dose that we adopted in our current study is reasonable.

**Figure 5:**
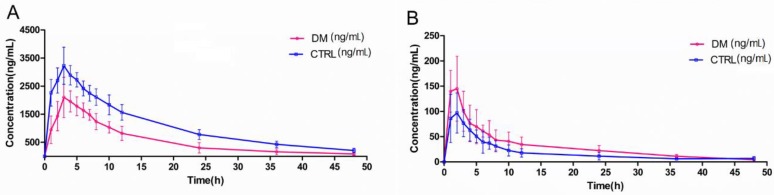

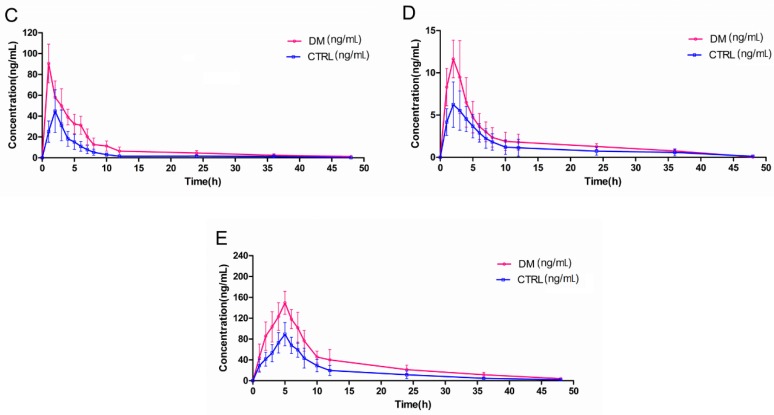
Plasma concentration–time curves for canagliflozin (**A**), M5 (**B**), M7 (**C**), M17 (**D**) and M9 (**E**) in rat plasma after oral administration (20 mg/kg) to DM and CTRL rats. Each point represents the mean ± SD (*n* = 5).

For the pharmacokinetic parameters of canagliflozin, including AUC_0–t_, AUC_0–∞_, CL_z_/F, and V_z_/F, there were significant differences between the CTRL and DM group rats (*p* < 0.05 or *p* < 0.01) (Figure 5) (Table 4). Meanwhile, the AUC_0–t_ and C_max_ values of the metabolites also showed significant differences (*p* < 0.05 or *p* < 0.01). The AUC values were decreased and the CL_z_/F and V values were increased, which indicates that the diabetic pathological state might reduce the absorption of canagliflozin and/or enhance its metabolism. In addition, the significantly increased AUC and C_max_ values of metabolites (M5, M7, M17 and M9) in DM rats were consistent with pharmacokinetics of canagliflozin. As such, care should be taken with regard to the efficacy of canagliflozin when its products are used in clinical treatments, especially when combined with CYP and UGT inducers or inhibitors.

We performed a study of the pharmacokinetics of canagliflozin and its metabolites between CTRL and DM rats (Figure 5). The CL_z_/F and AUC of the two groups displayed remarkable differences. Since the AUC is equal to the dose of administration divided by CL_z_/F, the changes in CL_z_/F would have had an influence on the values of AUC. The expression of drug-metabolizing enzymes and their activities greatly affected CL_z_/F, so we can conclude that diabetes may change the metabolic state of canagliflozin compared to healthy conditions (Table 4). According to previous studies, canagliflozin is mainly metabolized by UGT1A9 and UGT2B4, and partly through CYP3A4 in humans, but humans and animal species have different UGT gene systems. UGT1A9 is functional in humans, whereas rat Ugt1A9 is a pseudogene and Ugt1a7 compensates for the functions of Ugt1A9 in rats [27,28]. Shi R. et al. [29] and Xie H. et al. [30] have reported increased activities of UGT1A9 and Ugt1a7 under the pathology of diabetes, respectively. Moreover, Hu N, et al. [31] also reported the upregulated function and expression of CYP3A4 in type 2 diabetic rats. All these previous articles are consistent with the results of the pharmacokinetic behavior changes of canagliflozin and its metabolites in type 2 diabetic rats shown in the current study. Our group will continue to investigate the potential mechanisms of pharmacokinetic alternations of canagliflozin and its metabolites in diabetic pathological conditions.

In conclusion, our study successfully established a sensitive and effective UPLC-MS/MS quantification and semi-quantification method for canagliflozin and its metabolites and applied it to a pharmacokinetic study of type 2 diabetic rats. We investigated the validation of the method and found it to have sufficient specificity, accuracy and precision. In addition, canagliflozin in rat plasma was stable under the analytical conditions. Furthermore, we reported the pharmacokinetics and metabolic alternations of canagliflozin in the diabetic pathology in order to allow the drug to be used more safely, reasonably and effectively. The assay method used in the current study could provide a basis for canagliflozin metabolic research and the results of the pharmacokinetics.

## 3. Materials and Methods

### 3.1. Chemicals and Reagents

Canagliflozin (purity >98%) and empagliflozin (purity >98%) were purchased from Hubei Widely Chemistry Science and Technology Co., Ltd. (Wuhan, China). Heparin sodium and STZ (streptozocin) were purchased from Sigma Chemical Co., Ltd. (St Louis, MO, USA). A glucose test kit was purchased from Roche Co., Ltd. (Shanghai, China). Triglyceride and total cholesterol kits were from Beijing BHKT Clinical Reagent Co., Ltd. Rat insulin ELISA assay kits were supplied by Mercodia Co., Ltd. (Uppsala, Sweden). HPLC-grade methanol, acetonitrile, tert-butyl methyl ether and formic acid were purchased from Fisher Scientific Co., Ltd. (Pittsburgh, PA, USA). A Milli-Q water purification system was obtained from Millipore Corp Co., Ltd. (Bedford, MA, USA).

### 3.2. Apparatus

The UPLC-TOF-MS/MS system consisted of an API 5600 triple-TOF mass spectrometer (Sciex Co., Ltd., Framingham, MA, USA) and UPLC chromatographic analysis system (Shimadzu Co., Ltd., Kyoto, Japan). The UPLC-MS/MS system consisted of an API 5500 triple-quadrupole mass spectrometer (Sciex Co., Ltd., Framingham, MA, USA) and UPLC chromatographic analysis system (Shimadzu Co., Ltd., Kyoto, Japan). Chromatographic separation was performed using a Waters XBridge BEH C18 column (100 × 2.1 mm, 2.5 μm, Waters Co., Ltd., Dublin, Ireland).

### 3.3. UPLC-TOF-MS/MS Conditions

The chromatographic separation was performed on a C18 column, and the column temperature was maintained at 30 °C. The mobile phase consisted of 0.1% formic acid (A) and acetonitrile (B) with a flow rate of 0.3 mL/min. The gradient elution program started at 10% B, and ramped linearly to 90% B over 20 min. The sample injection volume was 3 μL.

For detection, the mass spectrometer was operated in the positive ion electrospray mode. A full scan was run with a mass range from *m*/*z* 100 to 1000 Da with a 0.25 s accumulation time. The conditions for the TOF-MS detector were as follows: ion spray voltage, 5500 V; turbo spray temperature, 550 °C; declustering potential (DP), 100 V; collision energy (CE), 35 eV. Nitrogen was used as the nebulizer and the auxiliary gas, and the nebulizer gas (gas 1), the heater gas (gas 2), and the curtain gas were set to 55, 55, and 35 psi, respectively. For the information-dependent data acquisition (IDA) criteria, the eight most intense fragment ions of each analyte that exceeded 100 cps value were selected for a product ion scan, and the ion scan ranged from 60 to 1300 Da with an 0.08 s accumulation time. The conditions of the product ions scan were as follows: collision energy (CE), 35 eV; collision energy scope (CES), 15 eV. High sensitivity mode was employed in the product ion scan.

### 3.4. UPLC-MS/MS Conditions

Separation was performed on a C18 column that was maintained at 30 °C and eluted on mobile phases of acetonitrile and 0.1% formic acid (*v*/*v* = 15/75) at a flow rate of 0.700 mL/min. The injection volume was kept at 3 μL. Elution followed a linear gradient, with acetonitrile content changing from 15% to 50% between 0 and 5.0 min. Acetonitrile content was then increased to 82% within 1 min. The total run time was 5.1 min.

The mass spectrometer was set to positive ionization mode. The MRM conditions were used to quantify the IS, canagliflozin and its metabolites. The Q1 mass, Q3 mass, declustering potential, entrance potential, collision energy and collision cell exit potential data are summarized in Table 5. The detection settings for mass spectrometer were as follows: temperature (TEM), 500 °C; ion spray voltage, 5500V; curtain gas, 20psi; gas 1, 55 psi and gas 2, 50 psi.

### 3.5. Preparation of Stock and Working Solutions, Calibration Standards and Quality Control Samples

Stock solutions of canagliflozin and empagliflozin (IS) were prepared in methanol at 1 mg/mL. The working solutions of canagliflozin were prepared by sequential dilution with methanol to final concentrations ranging from 100 ng/mL to 10,000 ng/mL. The IS working solution was diluted with methanol to give a final concentration of 5000 ng/mL. All solutions were stored at −20 °C and brought to room temperature before use. Calibration standard samples were prepared by spiking a working solution of canagliflozin and IS with the plasma followed by an extraction process to yield calibration standards of 100, 250, 500, 1000, 2500, 5000, 8000 and 10,000 ng/mL. Quality control (QC) samples were prepared as calibration standards to give nominal canagliflozin concentrations as the high (HQC), medium (MQC), and low (LQC) concentrations, including the lower limit of quantification (LLOQ).

### 3.6. Sample Extraction Procedure

To 100 μL of the standard and unknown plasma samples, 10 μL of the IS was added and mixed vigorously for 20 s. Five hundred milliliters of tert-butyl methyl ether was added, vortexed for 1 min and centrifuged for 12 min at 15,000 rpm. After transferring the supernatant to a fresh centrifuge tube, the plasma was extracted by tert-butyl methyl ether once again using same method. Then, the double supernatant was evaporated to dryness under a stream of nitrogen at 45 °C. One hundred milliliters of mobile phase was added to the resulting residue, vortexed for 1 min and 80 μL was transferred to UPLC sample vials.

### 3.7. Method Validation

The developed method was validated for linearity and sensitivity, specificity, accuracy, recovery and stability based on the guidelines for industry, Bioanalytical Method Validation, published by the US Food and Drug Administration. The calibration curve for canagliflozin ranging from 10 to 1000 ng/mL was generated by plotting the peak area ratios of the analyte to IS versus the nominal concentration and fitted by a weighted linear least-squares linear regression with a weighting factor of 1/*y*, where *y* was the peak area ratio of canagliflozin versus IS. The sensitivity of the method was evaluated in terms of LLOQ which was determined based on two criteria, as follows: (1) the analyte response at the LLOQ had to be at least 5 times larger than the blank response and (2) the analyte peak had to be identifiable, discrete, and reproducible with an accuracy (relative error) and precision (relative standard deviation, RSD) within 15%.

The specificity of the method was evaluated by comparing the chromatography of rat blank plasma samples with blank rat plasma spiked with standards at high, medium, low concentrations including LLOQ and rat plasma samples after oral administration of canagliflozin. The intra-day accuracy and precision were determined by analyzing five replicates of the QC samples at high, medium, and low concentrations within one day, while the inter-day accuracy and precision were conducted by determining five replicates of four levels of QCs on 3 separate days. The assay’s accuracy was represented by the nominal concentration. The precision was represented as the relative standard deviation (RSD) of the replicates. The matrix effect was determined by dividing the peak areas of canagliflozin spiked into extracted blank plasma by that of the analyte spiked in the mobile phase. The extraction recovery of canagliflozin and IS was assessed by comparing the peak areas of extracted plasma. In order to determine the stability of canagliflozin in rat plasma, bench-top stability, freeze–thaw stability, auto-injector stability and long-term stability studies were carried out with three replicates of the QC samples. In regard to bench-top stability during the handling process, the QC samples were prepared and kept at room temperature (25 °C) for 6 h. The freeze (−80 °C)–thaw (room temperature) stability of the analytes in rat plasma was tested via three freeze–thaw cycles. The stability of each sample was determined by analyzing the extracted QC samples after being kept in an auto-sampler at room temperature for 12 h. In regard to the storage stability, the QC samples were prepared and stored at −80 °C for 30 days.

The determination of metabolite concentrations was done by a semi-quantitative analysis method: the peak area of the metabolites was divided by the peak area of the original drug and then multiplied by the concentration of original drug.

### 3.8. STZ-Induced Type 2 Diabetic Rats

The type 2 diabetic model was developed using a combination of a high-fat diet (HFD) and low-dose STZ injection. Briefly, rats were randomized into CTRL (control) and DM (diabetes mellitus) groups. The CTRL rats received a standard diet, whereas DM rats were fed high-fat food, which consisted of 63.5–66.5% normal chow, 1% cholesterol, 0.5% sodium cholate, 10% yolk, 12–15% lard, and 10% sucrose. DM rats were given an intraperitoneal injection of STZ (35 mg/kg, dissolved in citrate buffer, pH 4.5) after 4 weeks of dietary alteration, and CTRL rats received an equivalent injection of citrate buffer only. The blood glucose concentrations, food intake, and body weights of the rats were monitored weekly. Rats with non-fasting blood glucose levels lower than 16.67 mmol/L (300 mg/dL) were excluded from the DM group. Biochemical parameters (serum triglycerides, serum total cholesterol, and insulin) were measured on day 28 after the injection of STZ. The homeostasis model assessment of insulin resistance (HOMA-IR) was calculated by a standard equation to examine insulin sensitivity. The following experiments were performed on day 35 after STZ injection.

### 3.9. Application to Pharmacokinetic Study in Type 2 Diabetic Rats

We performed the pharmacokinetic studies of canagliflozin after oral administration by gavage, and all rats used in this research were fasted for 12 h before dosing. CTRL and DM group rats (5 rats in each group) received a dose of canagliflozin at 20 mg/kg that was suspended in 0.5% sodium carboxymethylcellulose. A blood quantity of less than 0.3 mL was sampled via the oculi chorioideae vein after administration of canagliflozin at 1, 2, 3, 4, 5, 6, 7, 8, 10, 12, 24, 36 and 48 h under light ether anesthesia. All blood samples were immediately centrifuged at 5000 rpm for 10 min and the plasma was collected and frozen at −40 °C until analysis.

### 3.10. Data Analysis

The acquisition software used were Analyst TF 1.7.1 software and Analyst software (AB Sciex, Redwood city, CA, USA). The pharmacokinetic parameters were processed by non-compartmental analysis using DAS 2.1.1 software (Mathematical Pharmacology Professional Committee of China, Shanghai, China). Data are presented as means ± SDs. Statistical differences were evaluated by Student’s t-test. A *p*-value of less than 0.05 indicated a statistically significant difference (SPSS 18.0 software, SPSS Inc., Chicago, IL, USA).

## Figures and Tables

**Table 1:** Extraction recovery, intra-day and inter-day precision and accuracy data from UPLC-MS/MS for canagliflozin (*n* = 5).

Analyte	Concentration (ng/L)	Extraction Recovery (%)	Intra-Day		Inter-Day		Accuracy (%)
			Mean ± SD (ng/L)	RSD (%)	Mean ± SD (ng/L)	RSD (%)	
Canagliflozin	800	92.11 ± 5.31	786.44 ± 15.33	1.95	802.54 ± 13.22	1.65	6.12
	500	93.18 ± 3.11	503.19 ± 19.17	3.81	502.97 ± 17.79	3.53	4.08
	25	91.17 ± 2.14	26.44 ± 2.55	9.64	26.63 ± 1.95	7.32	3.26
	10	92.05 ± 1.51	11.56 ± 1.09	9.43	11.42 ± 1.22	10.68	4.13

**Table 2:** Stability of canagliflozin in rat plasma samples (*n* = 5).

Storage Conditions	Concentration (ng/L)	Mean ± SD	RSD %
Autosampler (4 °C) temperature for 24 h	25	25.72 ± 0.33	1.28
	500	503.15 ± 4.41	0.87
	800	806.81 ± 5.64	0.69
Room temperature (25 °C) for 24 h	25	26.21 ± 0.96	3.66
	500	504.45 ± 3.18	0.63
	800	807.28 ± 6.12	0.76
−80 °C temperature for 30 days	25	26.89 ± 0.85	3.16
	500	504.33 ± 2.14	0.42
	800	806.34 ± 3.51	0.44
Three freeze–thaw cycles (each at −80 °C for 24 h)	25	25.97 ± 1.02	3.93
	500	505.20 ± 4.12	0.82
	800	806.17 ± 4.19	0.52

**Table 3:** Biochemical parameters and alterations in experimental rats.

Parameters	CTRL	DM
Serum triglyceride (mmol/L)	2.53 ± 0.32	4.97 ± 0.61 *
Serum total cholesterol (mmol/L)	2.21 ± 0.65	5.12 ± 1.07 *
Initial serum glucose (mmol/L)	6.96 ± 0.69	7.12 ± 0.85
Final serum glucose (mmol/L)	7.01 ± 0.87	32.43 ± 3.23 **
HOMA-IR	6.02 ± 2.13	21.76 ± 11.14 **
Initial body weight (g)	221.46 ± 13.08	239.51 ± 15.02
Final body weight (g)	375.51 ± 29.67	387.31 ± 32.87

Data were expressed as (mean ± SD, *n* = 5), * *p* < 0.05, ** *p* < 0.01, versus CTRL rats.

**Table 4:** Non-compartmental pharmacokinetic parameters obtained for canagliflozin, M5, M7, M17 and M9 after oral administration (20 mg/kg) to DM and CTRL rats.

Parameters	Pharmacokinetic Parameters	CTRL	DM
Canagliflozin	AUC_0–t_ (ng·h/L)	51,988.40 ± 4162.72	27,116.66 ± 2694.322 **
	AUC_0–∞_ (ng·h/L)	56,314.67 ± 5095.46	28,423.95 ± 3072.73 **
	MRT_0–t_ (h)	14.08 ± 1.58	12.35 ± 2.77
	t_1/2z_ (h)	12.86 ± 2.64	10.24 ± 3.49 *
	T_max_ (h)	3.6 ± 0.89	3.20 ± 0.45
	CL_z_/F (L/h/kg)	357.46 ± 32.06	710.37 ± 77.98 **
	C_max_ (ng/L)	3310.00 ± 566.13	2226.00 ± 559.31 **
	V_z_/F (L/kg)	6600.39 ± 1372.71	10,508.36 ± 3544.44 **
M5	AUC_0–t_ (ng·h/L)	917.27 ± 227.42	1461.12 ± 371.70 **
	t_1/2z_ (h)	14.63 ± 18.29	13.32 ± 6.25
	T_max_ (h)	1.80 ± 0.84	1.67 ± 0.58
	C_max_ (ng/L)	126.42 ± 31.05	160.12 ± 54.34 *
M7	AUC_0–t_ (ng·h/L)	215.79 ± 26.95	501.35 ± 143.93 **
	t_1/2z_ (h)	10.04 ± 6.45	14.48 ± 4.86
	T_max_ (h)	2.02 ± 0.11	1.81 ± 0.12
	C_max_ (ng/L)	44.89 ± 20.5	90.53 ± 18.65 **
M17	AUC_0–t_ (ng·h/L)	58.80 ± 17.92	91.81 ± 24.22 **
	t_1/2z_ (h)	9.95 ± 3.57	9.02 ± 6.75
	T_max_ (h)	2.6 ± 0.89	2.33 ± 0.58
	C_max_ (ng/L)	7.27 ± 2.36	11.74 ± 2.37 *
M9	AUC_0–t_ (ng·h/L)	929.35 ± 226.29	1631.37 ± 131.63 **
	t_1/2z_ (h)	10.74 ± 1.63	12.78 ± 8.81
	T_max_ (h)	5.01 ± 0.11	4.75 ± 0.5
	C_max_ (ng/L)	97.49 ± 10.84	160.5 ± 10.48 *

AUC_0–t_, area under the curve of 0 to t time; AUC_0–∞_, area under the curve of 0 to infinity time; MRT_0–t_, mean residence time of 0 to t time; t_1/2z_, half time; T_max_, peak time; CL_z_/F, clearance divided by absorption fraction; C_max_, peak concentration; V_z_/F, apparent volume of distribution divided by absorption fraction. Data are expressed as means ± SDs, *n* = 5 rats * *p* < 0.05, ** *p* < 0.01, DM versus CTRL rats.

**Table 5:** Compound-dependent parameters in assay using UPLC-MS/MS.

Analyte	Q1 (*m*/*z*)	Q3 (*m*/*z*)	Declustering Potential (V)	Entrance Potential (V)	Collision Energy (V)	Collision Cell Exit Potential (V)
Canagliflozin	462.00	191.10	40.00	6.50	41.00	10.00
Empagliflozin (IS)	451.20	71.10	130.00	6.50	31.00	10.00
M5	638.00	191.10	50.00	6.50	30.00	10.00
M7	638.00	191.10	50.00	6.50	30.00	10.00
M17	638.00	191.10	50.00	6.50	30.00	10.00
M9	478.00	267.00	50.00	6.50	30.00	10.00

## Supplementary Materials

The Supplementary Materials are available online.
