# Supplementary material for: Plasma Pharmacokinetic Determination of Canagliflozin and Its Metabolites in a Type 2 Diabetic Rat Model by UPLC-MS/MS
Source: Molecules. 2018 May 20;23(5):1229. doi: 10.3390/molecules23051229 (PMC6100046; doi:10.3390/molecules23051229)
Supplement: Supplementary file 1 [file molecules-23-01229-s001.pdf]

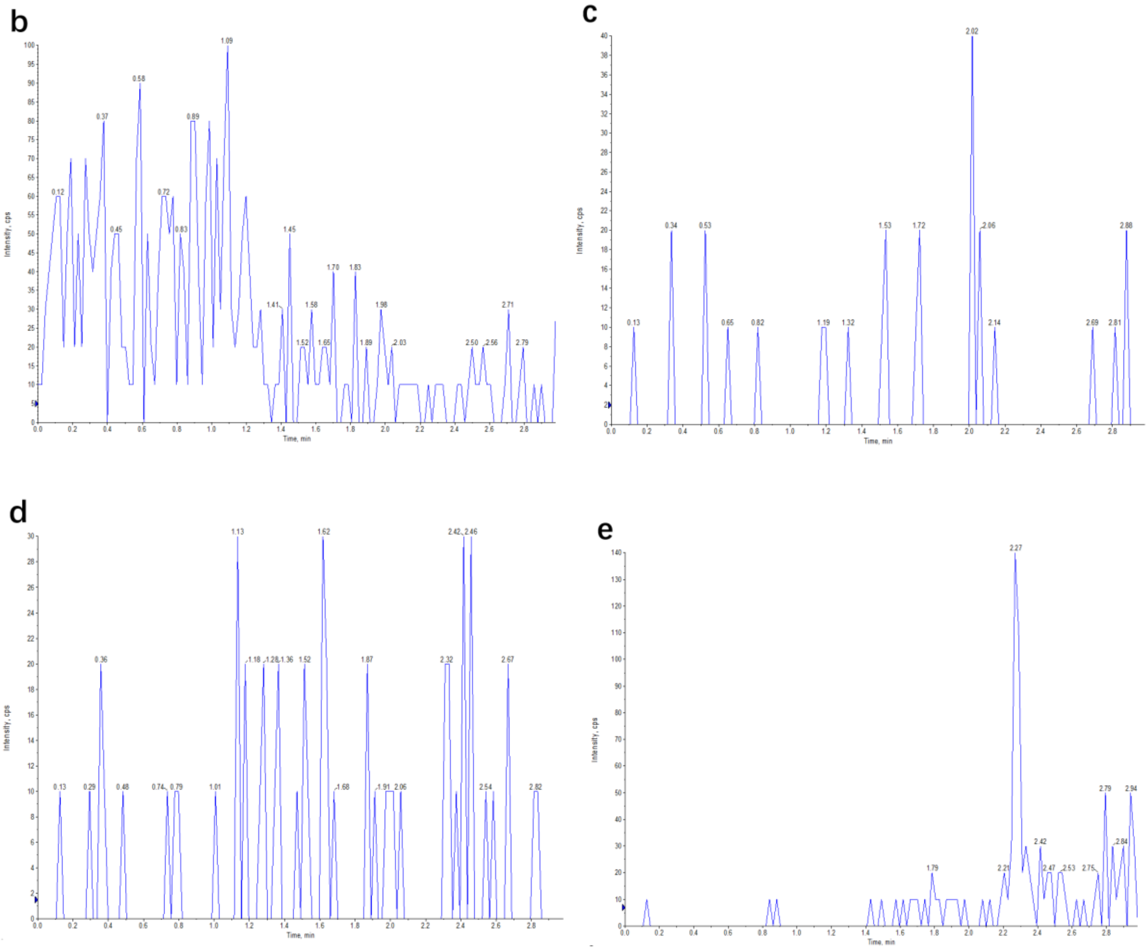

Figure S1. Figure b, c, d and e are the blank plasma figures of Figure B, C D and E under the corresponding conditions.
